# Interaction between SIRT1 and non-coding RNAs in different disorders

**DOI:** 10.3389/fgene.2023.1121982

**Published:** 2023-06-27

**Authors:** Soudeh Ghafouri-Fard, Hamed Shoorei, Bashdar Mahmud Hussen, Yadollah Poornajaf, Mohammad Taheri, Guive Sharifi

**Affiliations:** ^1^Department of Medical Genetics, Shahid Beheshti University of Medical Sciences, Tehran, Iran; ^2^Department of Anatomical Sciences, Faculty of Medicine, Birjand University of Medical Sciences, Birjand, Iran; ^3^ Clinical Research Development Unit of Tabriz Valiasr Hospital, Tabriz University of Medical Sciences, Tabriz, Iran; ^4^Department of Clinical Analysis, College of Pharmacy, Hawler Medical University, Erbil, Iraq; ^5^Faculty of Medicine, Birjand University of Medical Sciences, Birjand, Iran; ^6^ Institute of Human Genetics, Jena University Hospital, Jena, Germany; ^7^ Urology and Nephrology Research Center, Shahid Beheshti University of Medical Sciences, Tehran, Iran; ^8^ Skull Base Research Center, Loghman Hakim Hospital, Shahid Beheshti University of Medical Sciences, Tehran, Iran

**Keywords:** SIRT1, lncRNA, miRNA, circRNA, biomar

## Abstract

SIRT1 is a member of the sirtuin family functioning in the process of removal of acetyl groups from different proteins. This protein has several biological functions and is involved in the pathogenesis of metabolic diseases, malignancy, aging, neurodegenerative disorders and inflammation. Several long non-coding RNAs (lncRNAs), microRNAs (miRNAs) and circular RNAs (circRNAs) have been found to interact with SIRT1. These interactions have been assessed in the contexts of sepsis, cardiomyopathy, heart failure, non-alcoholic fatty liver disease, chronic hepatitis, cardiac fibrosis, myocardial ischemia/reperfusion injury, diabetes, ischemic stroke, immune-related disorders and cancers. Notably, SIRT1-interacting non-coding RNAs have been found to interact with each other. Several circRNA/miRNA and lncRNA/miRNA pairs that interact with SIRT1 have been identified. These axes are potential targets for design of novel therapies for different disorders. In the current review, we summarize the interactions between three classes of non-coding RNAs and SIRT1.

## Introduction

As a member of the sirtuin family, Sirt1 has a function in removal of acetyl groups from different proteins. This nicotinamide adenosine dinucleotide (NAD)-dependent deacetylase has several biological functions and is involved in the pathogenesis of metabolic diseases, malignancy, aging, neurodegenerative disorders and inflammation (Rahman and Islam, 2011). SIRT1 has a lot of substrates including a number of transcription factors. These transcription factors include p53, FoxO family, HES1, HEY2, PPARγ, CTIP2, p300, PGC-1α, and NF-κB (Haigis and Guarente, 2006; Michan and Sinclair, 2007; Yamamoto et al., 2007; Pillarisetti, 2008). The enzymatic reaction catalyzed by SIRT1 leads to generation of nicotinamide and transfer of the acetyl group of the substrate to cleaved NAD, producing a distinctive metabolite, namely, O-acetyl-ADP ribose (Pillarisetti, 2008).

SIRT1 has an important role in the regulation of energy homeostasis in response to accessibility to nutrients. In the liver tissue, SIRT1 enhances expression of the nuclear receptor PPARα, thus regulating lipid homeostasis. Deletion of Sirt1 in this tissue has been shown to impair PPARα signaling and decrease *ß*-oxidation of fatty acids, resulting in the development of hepatic steatosis, induction of inflammatory responses in liver, and endoplasmic reticulum stress (Purushotham et al., 2009).

In addition to the regulation of metabolic pathways, SIRT1 is involved in the carcinogenic processes. Its expression has been found to be increased in both hematological malignancies (Bradbury et al., 2005) and solid tumors (Huffman et al., 2007; Stünkel et al., 2007). Possibly acting as an oncogene, SIRT1 interacts with p53 and induces its deacetylation at its C-terminal Lys382 residue (Vaziri et al., 2001), thus inactivating this tumor suppressor.

In fact, SIRT1 is involved in a variety of human disorders including malignant and nonmalignant conditions. Recently, researchers have focused on identification of the interaction between non-coding RNAs and SIRT1 in these disorders. These investigations have led to identification of a number of long non-coding RNAs (lncRNAs), microRNAs (miRNAs) and circular RNAs (circRNAs) that regulate expression of SIRT1. In the current review, we provide an overview of these non-coding RNAs.

## SIRT1-interacting miRNAs

**FIGURE 1:**
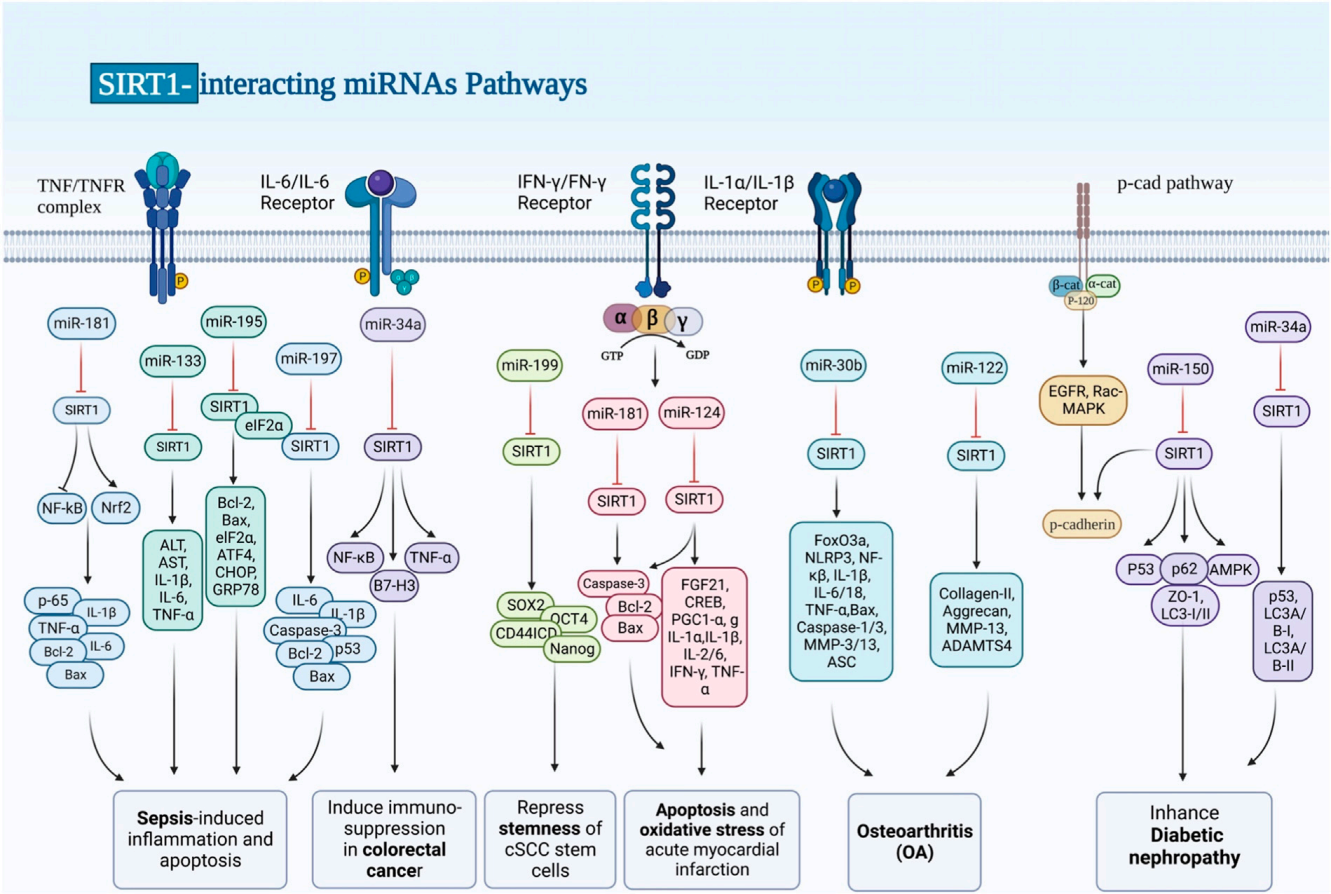
SIRT1 works with a lot of molecules, some of which are transcription factors. p53, the FoxO family, HES1, HEY2, PPAR, CTIP2, p300, PGC-1, and NF-B are all transcription factors. Dysregulation of SIRT1-targeting miRNAs plays a role in the pathogenesis of sepsis and its complications, chronic hepatitis, ischemia/reperfusion (I/R) injury to the liver and heart, cardiac fibrosis, myocardial infarction, osteoarthritis, diabetic nephropathy, and a number of malignant diseases like colorectal cancer.

A class of non-coding RNAs known as miRNAs regulate gene expression by binding to specific target genes in distinct pathways, thereby modulating the expression of various genes (Ghafouri-Fard et al., 2021a; Hussen et al., 2021; Hussen et al., 2022a). Mature miRNAs are formed by further processing of pre-miRNAs, which are formed from the transcribed nucleic acids that make up primary miRNAs. Several miRNAs have been shown to target SIRT1, thus regulating its expression. Dysregulation of SIRT1-targeting miRNAs is involved in the pathogenesis of sepsis and its complications, non-alcoholic fatty liver disease (NAFLD), chronic hepatitis, hepatic and myocardial ischemia/reperfusion (I/R) injury, cardiac fibrosis, heart failure, myocardial infarction, osteoarthritis, kidney injury, diabetic nephropathy, cerebral I/R Injury, spinal cord injury, epilepsy and a number of malignant conditions (Table 1; Figure 1). In sepsis, upregulation of miR-181a (Wu Z. et al., 2021), miR-133a (Chen L. et al., 2020) and miR-195 (Yuan et al., 2020) has been shown to lead to downregulation of SIRT1 and aggravation of inflammatory responses. miR-29a, miR-34a and miR-182 are among SIRT1-interacting miRNAs being involved in the pathogenesis of hepatic disorders. For instance, miR-29a via modulating the GSK-3β/SIRT1 could ameliorate mouse non-alcoholic steatohepatitis (Yang et al., 2020). Alterations in the miR-34a/SIRT1/FXR/p53 axis have been found to induce NAFLD in rats (Alshehri et al., 2021). Moreover, miR-34a via mediating the SIRT1/p53 axis could enhance liver fibrosis in patients with chronic hepatitis (Li X. et al., 2020).

**TABLE 1:** SIRT1-interacting miRNAs.

Type of diseases	miRNA	Sample	Cell line	SIRT1 expression	Targets and pathways	Discussion	Ref
Sepsis	miR-181a (Up)	-	RAW264.7	(Down)	Nrf2, p-65, NF-κβ, TNF-α, IL-1β, IL-6, Bcl-2, Bax	Inhibition of miR-181a via targeting SIRT1 by activating Nrf2 and inhibiting NF-κB could attenuate sepsis-induced inflammation and apoptosis	Wu et al. (2021a)
Sepsis	miR-133a (Up)	Serum samples: sepsis (n = 60), normal group (n = 30), C57BL/6J mice	RAW264.7	(Down)	ALT, AST, IL-1β, IL-6, TNF-α	miR-133a by targeting SIRT1 could aggravate inflammatory responses in sepsis	Chen et al. (2020a)
Sepsis	miR-195 (Up)	-	NCM460	(Down)	Bcl-2, Bax, elF2α, ATF4, CHOP, GRP78	miR-195 via targeting the SIRT1/eIF2α axis could enhance intestinal epithelial cell apoptosis	Yuan et al. (2020)
Sepsis	miR-197		H9c2	(Down)	Bcl-2, Bax, IL-6,	miR-197 by modulating SIRT1 could participate cardiomyocyte injury	Liu et al. (2022a)
					IL-1β, Caspase-3, p53		
Septic cardiomyopathy	miR-22 (-)	miR-22-flox mice, αMHC-Cre mice, littermates wild-type (WT) mice	Cardiomyocyte	(Down)	TNF-α, IL-6, IL-1β, LC3-I/II, p62, Atg7, Caspase-3/9, Bax, Bcl-2	Downregulation of miR-22 by targeting SIRT1 could alleviate septic cardiomyopathy	Wang et al. (2021a)
Non-alcoholic steatohepatitis (NAFLD)	miR-29a (-)	C57BL/6 mice	HepG2	(-)	GSK-3β, CD36, PERK, IRE1α, XBP1s, CHOP	miR-29a via modulating the GSK-3β/SIRT1 could ameliorate mouse non-alcoholic steatohepatitis	Yang et al. (2020)
NAFLD	miR-34a (Up)	Wistar rats	-	(Down)	FXR, p53, ALT, AST, γ-GGT,	Alteration of miR-34a/SIRT1/FXR/p53 axis could induce NAFLD in rats	Alshehri et al. (2021)
					TNF-α, IL-6,		
Chronic hepatitis C (CHC)	miR-34a (Up)	CHC (n = 41), healthy control samples (n = 18)	-	(Down)	p53, TBA, AST, ALT	miR-34a via mediating the SIRT1/p53 axis could enhance liver fibrosis in patients with chronic hepatitis	Li et al. (2020a)
Hepatic I/R Injury	miR-182 (-)	Black/Swiss mice, C57BL/6J WT mice	Hepatocyte	(Down)	XBP1, NLRP3, ALT, IL-1β, TNF-α, IL-18, Caspase-1	SIRT1 via modulating the miR-182-mediated XBP1/NLRP3 axis could alleviate hepatic IR injury	Li et al. (2021a)
Cardiac Fibrosis	miR-128 (Up)	C57BL/6 J mice	H9c2	(Down)	PIK3R1, p53, p62, Bcl-2, Bax,	Downregulation of miR-128 via targeting the SIRT1/PIK3R1 axis could ameliorate cardiac dysfunction	Zhan et al. (2021)
					Beclin-1, LC3-I/II, AKT, mTOR		
Congestive heart failure (CHF)	miR-22 (-)	C57BL/6 mice	Cardiomyocyte	(Down)	PGC-1α, TFAM, p62, LC3-I/II	Downregulation of miR-22 by targeting SIRT1/PGC-1α could alleviate CHF.	Wang et al. (2021b)
HF	miR-199a (Up)	C57Bl/6J mice	CMs, CFs, CECs	(Down)	P300, Yy1, sST2	miR199/SIRT1/P300 axis via upregulating the circulation of soluble sST2 isoform could modulate heart failure	Asensio-Lopez et al. (2021)
Myocardial I/R Injury	miR-29a (Up)	C57BL/J6	H9c2	(Down)	NLRP3, IL-1/6, IL-1β, TNF-α, eNOS, iNOS, Caspase-1	Downregulation of miR-29a by targeting SIRT1 and inhibiting NLRP3-mediated pyroptosis could ameliorate myocardial I/R Injury	Ding et al. (2020)
Cardiotoxicity	miR-200a-3p (Up)	Wistar rats	H9c2, 293T	(-)	PEG3, NF-κβ, Bax, Bcl-2, IKK, p65, IκBα	miR-200a-3p via modulating SIRT1/NF-κB axis and by targeting PEG3 could aggravate cardiotoxicity	Fu et al. (2021)
Acute myocardial infarction (AMI)	miR-181a-5p (-)	-	H9C2	(-)	Bcl-2, Bax,	miR-181a-5p via regulating SIRT1 could involve cardiomyocyte apoptosis induced by hypoxia–reoxygenation	Qi et al. (2020)
					Caspase-3		
AMI	miR-124-3p (Up)	SD rats	H9C2	(Down)	FGF21, CREB, PGC1-α, g IL-1α, IL-1β, IL-2/6, IFN-γ, TNF-α, Bax, Bcl-2, Caspase-3	miR-124-3p via targeting SIRT1 by modulation FGF21/CREB/PGC1α axis could regulate cell apoptosis and oxidative stress of acute myocardial infarction	Wei et al. (2021)
Osteoarthritis (OA)	miR-30b-5p (Up)	OA tissue samples (n = 40) and adjacent (n = 15) normal tissue samples, SD rats	HC-A,	(Down)	FoxO3a, NLRP3, NF-κβ, IL-1β, IL-6/18, TNF-α, Bax, Caspase-1/3, MMP-3/13, ASC	NF-κB-inducible miR-30b-5p via modulating SIRT1-FoxO3a-mediated NLRP3 inflammasome could aggravate joint pain	Xu et al. (2021a)
OA	miR-122 (Up)	OA tissue samples (n = 29), normal cartilage tissue samples (n = 29)	-	(Down)	Collagen-II, Aggrecan, MMP-13, ADAMTS4	miR-122 via targeting SIRT1 could regulate chondrocyte extracellular matrix degradation in osteoarthritis	Bai et al. (2020)
Kidney Injury	miR-34a (Up)	Kunming mice	-	(Down)	p53, TNF-α, IL-6, IL-1β, Caspase-9, Bax, Bcl-2	miR-34a/SIRT1/p53 axis could modulate kidney injury	Hao et al. (2021)
Acute kidney injury (AKI)	miR-183-3p (Up)	SD rats	NRK-52E	(Down)	PUMA, FOXO3a, TGF-β1, *a*-SMA, Vimentin,E-Cadherin	Depletion of miR-183-3p via the SIRT1/PUMA/FOXO3a axis could improve renal tubulointerstitial fibrosis after AKI.	Li et al. (2021b)
Diabetic nephropathy (DN)	miR-150-5p (Up)	(n = 60) diabetes mellitus patients, C57BL/6J mice	Podocyte	(Down)	p53, p62, AMPK, p-cadherin, ZO-1, LC3-I/II	Downregulation of miR-150-5p by targeting the SIRT1/p53/AMPK axis could ameliorate diabetic nephropathy	Dong et al. (2021)
DN	miR-34a (Up)	C57BL/6J mice	Podocyte	(Down)	p53, LC3A/B-I, LC3A/B-II	The p53/miR-34a/SIRT1 axis inhibition could ameliorate podocyte injury in DN.	Liang et al. (2021)
Cerebral I/R Injury	miR-19a/b-3p (Up)	SD rats	-	(Down)	FoxO3, SPHK1, NF-κβ p65, TNF-α, IL-6, IL-1β	miR-19a/b-3p via targeting the SIRT1/FoxO3/SPHK1 axis could promote inflammation during cerebral I/R injury	Zhou et al. (2021)
SCI	miR-324-5p (Up)	SD rats	PC12	(Down)	Bcl-2, Caspase-3, Bax, TNF-α, IL-1β	Silencing miR-324-5p by modulating SIRT1 could alleviate rats SCI.	Wang et al. (2021c)
CCIS	miR-34c-5p (Up)	SD rats	-	(Down)	TNF-α, IL-6, IL-1β, STAT3	Downregulation of miR-34c-5p via targeting the SIRT1/STAT3 axis could alleviate neuropathic pain	Mo et al. (2020)
Epilepsy	miR-135a-5p (Up)	-	BV2	(-)	Caspase-3/9	Downregulation of miR-135a-5p via targeting SIRT1 could protect glial cells against apoptosis in epilepsy	Wang et al. (2021d)
MDD	miR-138 (Up)	C57BL/6J mice	-	(Down)	PGC-1α, FNDC5, BDNF	miR-138 by targeting SIRT1 could enhance depressive-like behaviors in the hippocampus	Li et al. (2020b)
Migraine	miR-34a-5p (-)	SD rats	trigeminal ganglionic cells	(-)	COX2, PGE2, p65, NF-κβ, IL-1β, IL-13	miR-34a-5p via inhibiting SIRT1 could enhance the IL-1β/COX2/PGE2 axis and stimulate the release of CGRP in trigeminal ganglion neurons in rats	Zhang et al. (2021a)
DFUs	miR-489-3p (-)	SD rats	HUVECs	(-)	VEGF, Bcl-2, Bax, Caspases-3/9, PI3K, AKT, eNOS, iNOS	Alteration in miR-489-3p/SIRT1 axis could enhance wound healing in DFU.	Huang et al. (2021a)
DR	miR-221 (Up)	-	hRMEC	(Down)	Nrf2, Caspase-3 Bax, Bcl-2, Keap-1	Overexpression of miR-221 via inhibiting SIRT1 could enhance apoptosis of hRMEC.	Chen et al. (2020b)
ALI	miR-146a-3p (Up)	SD rats	BEAS-2B	(Down)	NF-κβ, TNF-α, IL-1β, IL-4, IL-6, IL-10	Depletion of miR-146a-3p via upregulating SIRT1 and mediating NF-κB could attenuate ALI.	Yang and Li (2021)
UUO	miR-155-5p (Up)	-	NRK-49F	(Down)	α-SMA, Collage-I, Fibronectin	miR-155-5p via modulating SIRT1 promotes renal interstitial fibrosis	Wang et al. (2021e)
-	miR-217 (Up)	-	HUVECs	(-)	p53, SA-β-gal	miR-217 via modulating the SIRT1/p53 axis could enhance endothelial cell senescence	Wang et al. (2021f)
-	miR-204-5p (-)	C57BL/6J	HC11	(-)	PPARγ	miR-204-5p by targeting SIRT1 could enhance lipid synthesis in mammary epithelial cells	Zhang et al. (2020a)
-	miR-128-3p (Up)	-	BMSCs	(-)	IL-6, IL-1β, MMP-9, MCP-1	MiR-128-3p by regulating SIRT1 expression could mediate inflammatory responses in BMSCs	Wu et al. (2020)
-	miR‐34a‐5p, miR‐34a‐3p (-)	Human submandibular gland tissue samples (n = 114), human parotid gland tissue samples (n = 114), serum samples (n = 114), SD rats	SMG‐C6,	(-)	CTRP6, AMPK, TNF‐α, Bcl-2, Bax Caspase-3/8/9/12, Cytochrome-C,	CTRP6 via targeting the AMPK/SIRT1 axis by modulating miR‐34a‐5p expression could attenuate TNF‐α‐induced apoptosis	Qu et al. (2021)
-	miR-146a-5p (Up)	(n = 45) bone tissue samples, KO mice	MC3T3-E1	(-)	Collagen-I	miR-146a-5p via targeting SIRT1 could regulate bone mass	Zheng et al. (2021)
PCa	miR-373 (-)	-	AsPC-1,	(-)	PGC-1α, NRF2, Bax, Bcl-2, Caspase-3/8/9, PARP, eNOS, iNOS	miR-373 via modulating the SIRT1/PGC-1α/NRF2 axis could suppress cell proliferation in pancreatic cancer cells	Yin et al. (2021)
			PANC-1				
CRC	miR-34a (Up)	CRC tissue and ANT samples, DAB1/J mice, NOD-SCID mice	HCT-8,	(-)	NF-κβ, p65, B7-H3, TNF-α	miR-34a via modulating the SIRT1/NF-κB and B7-H3/TNF-α axis could induce immunosuppression in colorectal cancer	Meng et al. (2021)
			HCT-116,				
			CHO, PBMCs				
cSCC	miR-199a-5p (Down)	BALB/c nude mice	A431, NHSF	(Up)	CD44ICD, OCT4, SOX2, Nanog	miR-199a-5p by targeting SIRT1 and CD44ICD cleavage signaling could repress stemness of cSCC stem cells	Lu et al. (2020)

miR-128 has been shown to be involved in the pathogenesis of chronic angiotensin II infusion-induced cardiac remodeling through modulation of SIRT1. Silencing this miRNA in the heart tissues of mice could ameliorate angiotensin II-induced cardiac dysfunction, hypertrophy, fibrosis and oxidative stress damage. Angiotensin II could induce upregulation of miR-128 in cell culture. Treatment of cells with miR-128 antagomir could attenuate angiotensin II -induced apoptosis and oxidative damage possibly through targeting the SIRT1/p53 pathway. Suppression of this miRNA could also activate PIK3R1/Akt/mTOR pathway, restrain angiotensin II-induced autophagy in cardiomyocytes, and mitigate oxidative stress and apoptosis (Zhan et al., 2021).

SIRT1-interacting miRNAs are also involved in the pathogenic processes in the acute myocardial infarction. Suppression of miR-29a has been shown to protect against myocardial I/R injury through influencing expression of SIRT1 and subduing oxidative stress and NLRP3-associated pyroptosis (Ding et al., 2020). In addition, miR-200a-3p has been found to aggravate doxorubicin-induced cardiotoxic effects through targeting PEG3 via SIRT1/NF-κB signaling pathway (Fu et al., 2021). miR-181a-5p is another miRNA which participates in the cardiomyocyte apoptosis induced by hypoxia–reoxygenation via regulation of SIRT1 (Qi et al., 2020). Moreover, an experiment in an animal model of acute myocardial infarction has shown that miR-124-3p targets SIRT1 to influence cell apoptosis, inflammatory responses, and oxidative stress through regulation of the FGF21/CREB/PGC1α axis (Wei et al., 2021). Besides, miRNAs that modulate expression of SIRT1 can affect pathogenesis of heart failure. For instance, downregulation of miR-22 by targeting SIRT1/PGC-1α could alleviate this disorder (Wang et al., 2021b). Finally, miR199/SIRT1/P300 axis has apotential function in the patheticlogy of this disorder (Asensio-Lopez et al., 2021).

Lastly, three SIRT1-interacting miRNAs have been revealed to participate in the carcinogenesis. miR-373 is a tumor suppressor miRNA that inhibits proliferation of pancreatic cancer cells through influencing activity of SIRT1/PGC-1α/NRF2 axis (Yin et al., 2021). On the other hand, miR-34a acts as an immunosuppressive miRNA in colorectal cancer via regulation of SIRT1/NF-κB/B7-H3/TNF-α axis (Meng et al., 2021). Lastly, miR-199a-5p has a role in repression of stemness of squamous cell carcinoma cells through influencing activity of SIRT1 and CD44ICD cleavage signaling (Lu et al., 2020).

## SIRT1-interacting circRNAs

**FIGURE 2:**
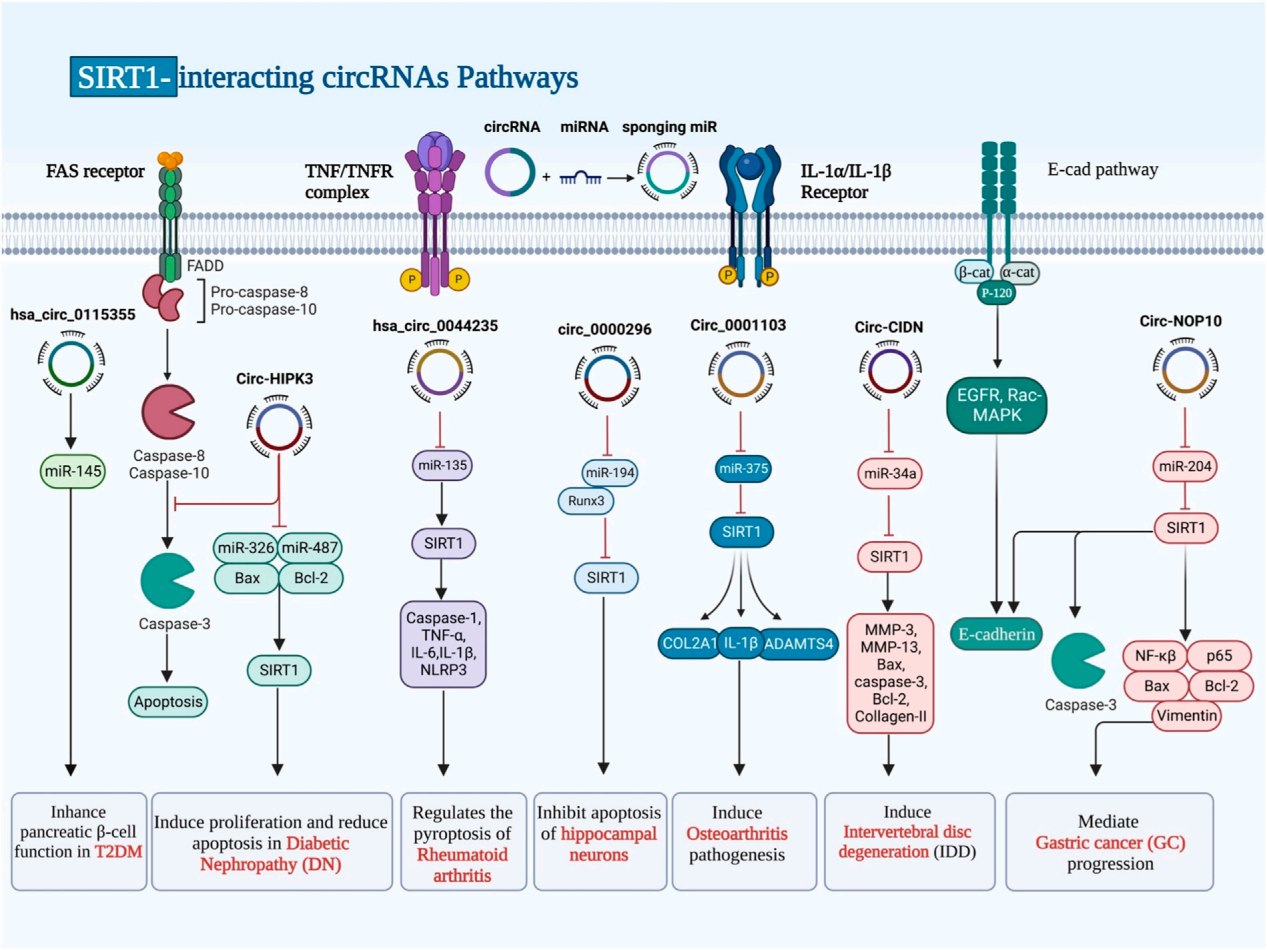
CircRNAs have been proven to serve as molecular sponges for miRNAs, thereby influencing the expression of miRNA targets. SIRT1 has been found to be the target of miRNA genes, which were already being sponged by different types of circRNAs that prevented or enhanced gene expression.

Circular RNAs (CircRNAs) are common in all animals, from viruses to mammals. They are single-stranded, endogenous covalently closed RNA molecules with highly stability. The biosynthesis, regulation, localization, destruction, and modification of circRNAs have all seen great progress (Sayad et al., 2022). CircRNAs play a role in a wide range of human disorders, particularly malignancies (Ghafouri-Fard et al., 2021b; Ghafouri-Fard et al., 2022). The impact of SIRT1-interacting circRNAs in the regulation of SIRT1 has been assessed in diabetes and its complications, rheumatoid arthritis, chronic cerebral ischemia, osteoarthritis, intervertebral disc degeneration as well as malignant disorders, particularly glioma (Table 2). All of these circRNAs have been shown to act as molecular sponges for miRNAs to subsequently affect expression of miRNAs targets (Figure 2). For instance, hsa_circ_0115355 has been found to regulate activity of miR-145/SIRT1 axis, thus enhancing function of pancreatic *ß* cells in patients with type 2 diabetes mellitus (Dai et al., 2022). CircHIPK3 is another circRNA which participates in the pathogenesis of diabetic complications. Expression of this circRNA has been significantly reduced in HK-2 cells following exposure with high glucose. Forced upregulation of circHIPK3 could reverse high glucose-induced pathologic events in HK-2 cells. SIRT1 has been found to be the target of miR-326 and miR-487a-3p, two downstream genes of circHIPK3. Silencing of these two miRNAs could induce proliferation and decrease apoptosis in high glucose-induced HK-2 cells. Taken together, upregulation of circHIPK3 can reduce the effects of high glucose in HK-2 cells via sponging miR-326 or miR-487a-3p and influencing expression of SIRT1 (Zhuang et al., 2021).

**TABLE 2:** SIRT1-interacting circRNAs.

Type of diseases	Circular-RNAs	Sample	Cell line	SIRT1 expression	Targets and pathways	Discussion	Ref
T2DM	hsa_circ_0115355 (Down)	Serum samples of T2DM patients (n = 20)	INS-1	(Down)	miR-145	hsa_circ_0115355 via targeting the miR-145/SIRT1 axis could enhance pancreatic *ß*-cell function	Dai et al. (2022)
DN	HIPK3 (Down)	-	HK-2	(Down)	miR-326,	Circ-HIPK3 via modulating the miR-326/miR-487a-3p/SIRT1 axis could alleviate high glucose toxicity to HK-2 Cells	Zhuang et al. (2021)
					miR-487a-3p,		
					Caspase-3,		
					Bax, Bcl-2		
RA	hsa_circ_0044235 (Down)	Serum samples of RA (n = 48), healthy control group (n = 36), DBA/1 J mice	FLSs	(Down)	miR-135b-5p, Caspase-1,	hsa_circ_0044235 could regulate pyroptosis via modulating miR-135b-5p-SIRT1 axis	Chen et al. (2021)
					TNF-α, IL-6,		
					IL-1β, NLRP3		
CCI	circ_0000296 (Down)	C57BL/6J mice	HT22	(Down)	miR-194-5p, Runx3	Upregulation of circ_0000296 via miR-194-5p/Runx3 axis could increase transcription of SIRT1 and inhibit apoptosis of hippocampal neurons	Huang et al. (2021b)
OA	Circ_0001103 (Down)	OA samples (n = 30), normal tissues (n = 10), OA serum (n = 10) and normal (n = 10) samples	Chondrocyte	(Down)	miR-375, IL-1β, COL2A1, ADAMTS4	Circ_0001103 via targeting miR-375 by upregulating SIRT1 could alleviate IL-1β-induced chondrocyte cell injuries	Zhang et al. (2021b)
IDD	CIDN (Down)	IDD tissue samples (n = 30) and healthy control tissues (n = 50), SD rats	NP	(Down)	miR-34a-5p,	Circ-CIDN via the miR-34a-5p/SIRT1 axis could mitigate compression loading-induced damage	Xiang et al. (2020)
					MMP-3/13, Bax, caspase-3, Bcl-2,		
					Collagen-II		
GC	NOP10 (Up)	10 pairs of GC and ANT samples	GES-1, AGS,	(Up)	miR-204, NF-κβ, E-cadherin, p65, Vimentin, Bcl-2, Caspase-3, Bax	Circ-NOP10 by regulating the miR-204/SIRT1 axis could mediate gastric cancer progression	Xu et al. (2021b)
			MNK-45,				
			HGC-27,				
			BGC-823				
Glioma	Circ-0082374 (Up)	glioma samples (n = 42), non-cancer tissue samples (n = 28), BALB/c nude mice	A172, BT325,	(-)	miR-326,	Knockdown of Circ-0082374 by modulating the miR-326/SIRT1 axis could inhibit viability, migration, invasion, and glycolysis of glioma cells	Wang et al. (2020)
			LN229, U251,		MMP-9,		
			SHG44, HA1800		E-cadherin, Vimentin		

Hsa_circ_0044235 is another circRNA which has been shown to be downregulated in patients with rheumatoid arthritis (RA). Downregulation of this circRNA has been correlated with low levels of SIRT1 expression in these patients. Overexpression of hsa_circ_0044235 could attenuate joint inflammation, cell apoptosis, and joint injury, and reduce NLRP3-mediated pyroptosis but increasing SIRT1 expression. Upregulation of this circRNA could also inhibit caspase-1 content. Mechanistically, hsa_circ_0044235 increases expression of SIRT1 through sponging miR-135b-5p (Chen et al., 2021).

CircularNOP10 and circ0082374 are two putative oncogenic circRNAs that regulate expression of SIRT1. CircularNOP10 has a role in induction of progression of gastric cancer through regulation of miR-204/SIRT1 pathway (Xu J. et al., 2021). In glioma cells, circ0082374 has a role in induction of cell viability, migration, invasion and glycolysis through regulation of miR-326/SIRT1 axis (Wang et al., 2020).

## SIRT1-interacting lncRNAs

Transcripts larger than 200 nt are known as long non-coding RNAs (lnRNAs), which cannot code for proteins and may possess small open reading frames (ORFs). Because they interact with various proteins, mRNAs and DNA sequences, lncRNAs play significant roles in a number of disorders (Sabaie et al., 2021; Hussen et al., 2022b). GAS5, LincRNA‐p21, MCM3AP-AS1, TUG1, SNHG7, SNHG8, SNHG10, SNHG15, Oip5-as1, ILF3-AS1, ANRIL, UCA1 and KCNQ1OT1 are examples of lncRNAs that regulate expression of SIRT1 through sponging miRNAs. These lncRNAs can affect pathogenesis of RA, atherosclerosis, sepsis-associated renal injury (SARI), diabetic nephropathy, ischemic stroke and a number of malignant conditions (Table 3). For instance, GAS5 via regulating the miR-222-3p/Sirt1 axis could alleviate RA (Yang et al., 2021). Moreover, GAS5 via inhibiting the miR-579-3p and activating the SIRT1/PGC-1α/Nrf2 axis could reduce cell pyroptosis in SARI (Ling et al., 2021). In the context of osteoarthritis, MCM3AP-AS1 via modulating the miR-138-5p/SIRT1 axis could protect chondrocytes from IL-1β-induced inflammation (Shi et al., 2021).

**TABLE 3:** SIRT1-interacting lncRNAs.

Type of diseases	LncRNA	Sample	Cell line	SIRT1 expression	Target and pathways	Discussion	Ref
RA	GAS5 (Down)	Serum samples of RA patients (n = 35) and Serum samples of healthy control (n = 35)	RA-FLSs	(Down)	miR-222-3p, TNF-α,IL-1β, IL-6, Bcl-2, Bax, Caspase-3/9	GAS5 via regulating the miR-222-3p/Sirt1 axis could alleviate RA.	Yang et al. (2021)
Atherosclerosis	LincRNA‐p21 (Down)	Serum samples of AS patients (n = 25), C57BL/6 mice	HAECs,	(Down)	miR-221, Pcsk9, Caspase-3, Bcl-2, Bax	LncRNA-p21 via modulating the miR-221/SIRT1/Pcsk9 axis could alleviate atherosclerosis progression	Wang et al. (2021g)
			293T				
SARI	GAS5 (Down)	C57BL/6 mice	HK-2	(Down)	miR-579-3p, Nrf2, IL-1β, IL-18, NLRP3, PGC-1α, Caspase-1,	GAS5 via inhibiting the miR-579-3p by activating the SIRT1/PGC-1α/Nrf2 axis could reduce cell pyroptosis in SARI.	Ling et al. (2021)
OA	MCM3AP-AS1 (Down)	30 pairs of OA and ANTs	CHON-001, ATDC5	(Down)	miR-138-5p,	MCM3AP-AS1 via modulating the miR-138-5p/SIRT1 axis could protect chondrocytes from IL-1β-induced inflammation	Shi et al. (2021)
					IL-6, IL-8,		
					TNF-α		
Sepsis	TUG1 (Down)	C57BL/6 mice	RAW264.7	(Down)	miR-9-5p, TNF-α, MCP-1, IL-6, IL-10, iNOS, Arg-1,	TUG1 via impairing miR-9-5p targeted SIRT1 inhibition could confer anti-inflammatory macrophage polarization in sepsis effects	Ma et al. (2021)
DN	TUG1 (Down)	-	HK-2	SIRT1 (Down)	miR-29c-3p, ERS, Bax, Bcl-2, caspase-3/12, GRP78, CHOP, PERK, eIF-2α	The TUG1/miR-29c-3p/SIRT1 axis could regulate endoplasmic reticulum stress-mediated cell injury in DN.	Wang et al. (2021h)
Ischemic Stroke	SNHG8 (-)	C57BL/6 mice	BMEC	(Down)	miR‐425‐5p, NF‐κβ, caspase-3, ZO-1, Occludin, TNF-α, IL-1β, IL-6	SNHG8 via regulating miR‐425‐5p mediated SIRT1/NF‐κβ axis could attenuate blood-brain barrier damage	Tian et al. (2021)
Ischemic Stroke	SNHG7 (Down)	C57BL/6 mice	PC12,	(Down)	miR-9	SNHG7 by targeting the miR-9/SIRT1 axis could alleviate damage in PC12 Cells	Zhou et al. (2020)
Cerebral I/R Injury	SNHG15 (Up)	-	SH-SY5Y	(-)	miR-141, TNF-α, IL-1β, IL-6, iNOS, p65	SNHG15 by targeting the miR-141/SIRT1 axis could enhance oxidative stress damage	Kang et al. (2021)
Myocardial I/R Injury	Oip5-as1 (Down)	SD rats	NRVMs,	(Down)	miR-29a, AMPK, PGC1α, LDH, ROS, Bax, Bcl-2, Cyt-c, caspase-3, 15-F2t-isoprostane, SOD, GPx	Oip5-as1 via activating the SIRT1/AMPK/PGC1α axis by sponging miR-29a could attenuate myocardial I/R injury	Niu et al. (2020)
			H9c2				
MI	ILF3-AS1 (Down)	-	H9c2,	(-)	miR-212-3p, PI3K, AKT, Bcl-2, Bax, caspase-3/9	ILF3-AS1 via targeting the miR-212-3p/SIRT1 axis and the PI3K/Akt pathway could regulate MI.	Zhang et al. (2020c)
			293T				
AMI	ANRIL (Up)	-	H9c2	(-)	miR‐7‐5p, Bcl-2, Bax, Caspase-3/9, HIF-1α	ANRIL via targeting the miR‐7‐5p/SIRT1 axis could protect H9c2 cells against hypoxia‐induced injury	Shu et al. (2020)
Diabetes	TUG1 (Down)	C57BL/6J mice	3T3-L1	(Down)	miR-204, AMPK, ACC, ATGL, PGC-1α, PPARα, UCP-1	TUG1 via targeting SIRT1 by regulating miR-204 could enhance brown remodeling of white adipose tissue in diabetic mice	Zhang et al. (2020d)
NSCLC	SNHG10 (Down)	60 pairs of NSCLC and ANT samples	H1581, H1703	(-)	miR-543	SNHG10 via sponging miR-543 could upregulate tumor suppressive SIRT1 in NSCLC.	Zhang et al. (2020b)
HCC	SNHG7 (Up)	25 pairs of HCC and ANT samples	THLE-3,	(Up)	miR-34a, NLRP3, Caspase-1, IL-1β	SNHG7 via targeting the miR-34a/SIRT1 axis could inhibit NLRP3-dependent pyroptosis	Chen et al. (2020c)
			293T, HepG2,				
			SK-hep-1				
CRC	GAS5 (Down)	75 pairs of CRC and ANT samples, Wistar rats	HT29,	(Up)	miR-34a, mTOR, LC3 I/II. Beclin-1, Bcl-2, Bax	GAS5 via targeting the miR-34a/mTOR/SIRT1 axis could inhibit malignant progression in CRC.	Zhang et al. (2021c)
			HCT116,				
			SW480,				
			SW620				
AML	UCA1 (Up)	Serum samples: AML (n = 27), normal (n = 9)	KG‐1a, THP‐1,	(-)	miR‐204, Caspase-3, iNOS, COX-2	Silencing UCA1 via targeting miR‐204 by repressing SIRT1 could accelerate apoptosis in pediatric AML.	Liang et al. (2020)
			HS‐5				
RB	KCNQ1OT1 (UP)	3 pairs of RB and ANTs, nude mice	hTERT RPE-1, Y79, WERI-Rb-1	(Down)	miR-124,	KCNQ1OT1 by targeting the miR-124/SP1 axis could modulate RB cell proliferation and invasion	Zhang et al. (2021d)
					SP1, Cyclin-D1,		
					Caspase-3, Vimentin,		
					E/N-cadherin,		

SIRT1-interacting lncRNAs have also been shown to affect pathogenesis of malignant conditions. For instance, SNHG10 has been found to sponge miR-543 in non small cell lung cancer (Zhang Z. et al., 2020). Moreover, SNHG7 has been demonstrated to inhibit NLRP3-associated pyroptosis through regulating miR-34a/SIRT1 axis in liver cancer (Chen Z. et al., 2020). GAS5 can inhibit malignant progression of colorecatl cancer cells through regulating macroautophagy and forming a negative feedback loop with the miR-34a/mTOR/SIRT1 axis (Zhang HG. et al., 2021). On the other hand, UCA1 has a role in induction of cell proliferation and suppression of apoptosis through affecting expression of SIRT1 and miR‐204 in pediatric AML (Liang et al., 2020). The known interactions that SIRT1 has with a variety of lnRNAs are illustrated in Figure 3.

**FIGURE 3:**
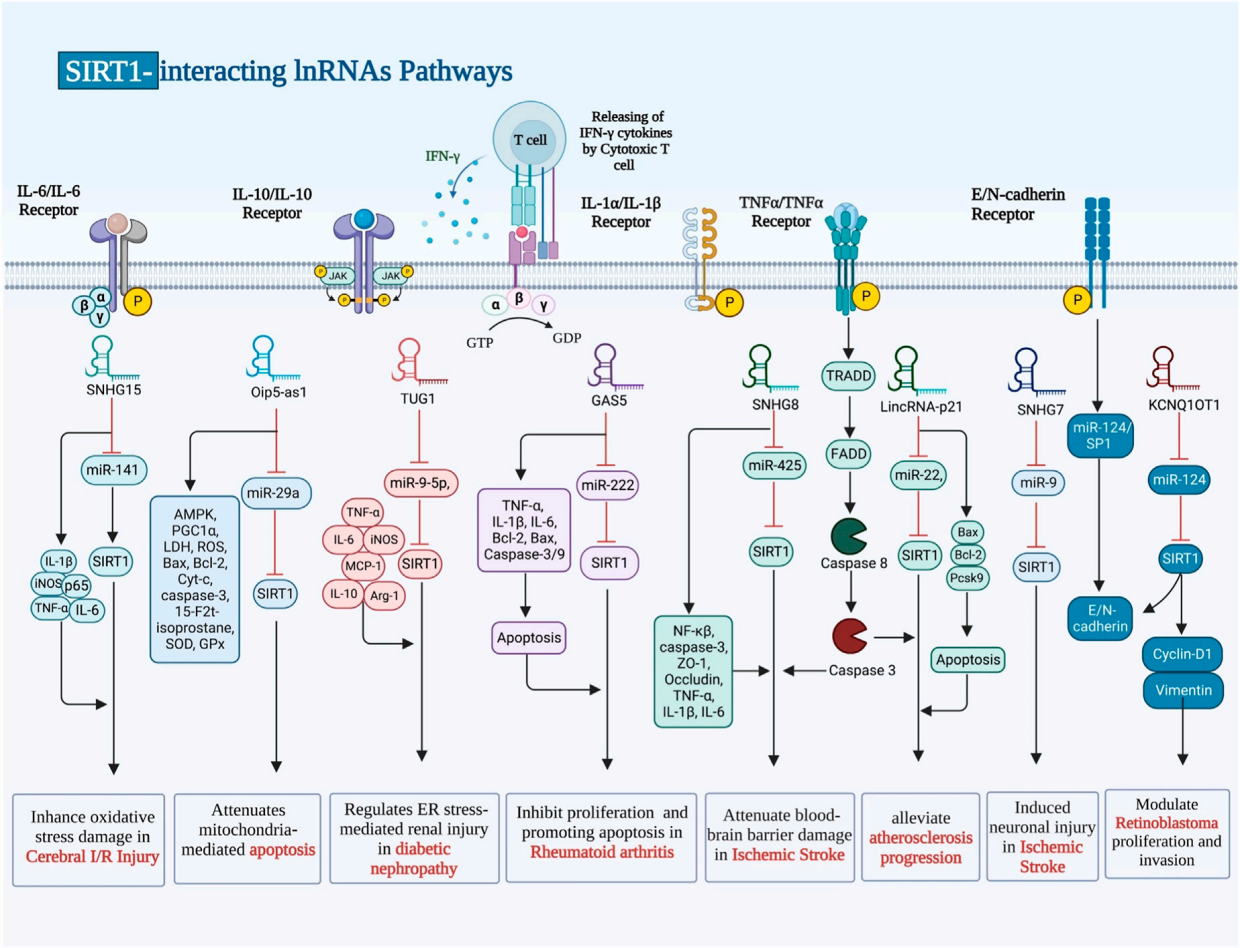
There are numerous ways in which SIRT1 and the other lnRNAs interact, and it has been demonstrated that these interactions have an impact on pathogenesis conditions.

A number of therapeutic agents such as anthocyanins, ginsenoside-R3, dexmedetomidine hydrochloride, berberine, sorafenib, 17β-Estradiol, phenylpyridinium, tetrahydroxy stilbene glycoside, cisplatin, resveratrol, sulforaphane and liraglutide have been found to affect expression of non-coding RNAs/SIRT1 axes (Table 4). For instance, experiments in animal model of asthma have shown that anthocyanins suppresses inflammatory responses in airways through decreasing activity of NF-κB pathway via the miR-138-5p/SIRT1 axis (Liu Y. et al., 2022). Moreover, ginsenoside Rg3 can alleviate sepsis-related hepatic injury through modulation of TUG1/miR-200c-3p/SIRT1 axis (Wu P. et al., 2021). TUG1/miR-194/SIRT1 axis has been found to be targeted by dexmedetomidine hydrochloride to inhibit hepatocytes apoptosis and inflammatory responses (Gu et al., 2021). Additionally, the effects of berberine in amelioration of hepatic insulin resistance have been revealed to be mediated through regulation of miR-146b/SIRT1 axis (Sui et al., 2021).

**TABLE 4:** Effects of drugs on SIRT1-interacting ncRNAs.

Type of diseases	Drug	Non-coding RNAs	Sample	Cell line	SIRT1 expression	Target	Discussion	Ref
Asthma	Anthocyanins (Anth)	miR-138-5p (Up)	Balb/c mice; treated with 250 mg/kg Anth before each atomization for 1 h	HBE; treated with 10 μg/mL Anth for 1 h	(-)	NF-κβ p65,	Anth via targeting the miR-138-5p/SIRT1 axis by downregulating NF-κβ could inhibit airway inflammation in asthmatic mice	Liu et al. (2022b)
						IL-4/5/13,		
						IFN-γ		
Sepsis	Ginsenoside-R3 (Rg3)	TUG1 (-), miR-200c-3p	C57BL/6 mice; treated with 20 mg/kg Rg3, I.P, for 1 h	Hepatocyte; pretreated with 25 μM Rg3 for 6 h	(Down)	LC3-I/II, p62, Beclin-1,	Rg3 by modulating the TUG1/miR-200c-3p/SIRT1 axis could alleviate septic liver injury	Wu et al. (2021b)
						PGC1-α,		
						AMPK		
LI	Dexmedetomidine hydrochloride (DEX)	TUG1 (-), miR-194	-	WRL-68; pretreated with 0.01, 0.1, and 1, 5 nM DEX for 1 h	(Down)	Bax, Bcl-2,	DEX by activating the TUG1/miR-194/SIRT1 axis could inhibit hepatocyte inflammation and apoptosis	Gu et al. (2021)
						TNF-α,		
						IL-1β, IL-6		
Insulin resistance	Berberine (BBR)	miR-146b (-)	C57BL/6J mice; treated with 5, 10 mg/kg/day, I.P, for 4 weeks,	HepG2; treated with 5–30 µM BBR for 24h and 48 h	(Down)	FOXO1	BBR by regulating the miR-146b/SIRT1 axis could ameliorate hepatic insulin resistance	Sui et al. (2021)
Liver cancer	Sorafenib	miR-425 (-)	TCGA and GEO databases	HepG2, PLC, Hep3B, Huh7, MIHA; treated with 10 µM for 48 h	(-)	LC3-I/II,	miR-425 via SIRT1 to promote sorafenib resistance could regulate lipophagy in liver cancer	Sun et al. (2021)
						ATGL		
PMOP	17β-Estradiol (E2)	H19 (Down), miR-532-3p	Bone tissue (n = 10), serum samples (n = 10), control group (n = 10), Wistar rats; treated with 0.5 mg/kg/day E2 subcutaneously	BMSCs; treated with 10^–7^ M E2 for 14 days	(Down)	ALP, RUNX2,	E2 via targeting the miR-532-3p/SIRT1 axis could enhance the expression of H19 to regulate osteogenic differentiation	Li et al. (2021c)
PD	Phenylpyridinium (MPP)	miR-132 (-)	FVB littermate wild-type mice	SH-SY5Y; treated with 1.25 and 2.5 mM MPP, for 12, 24, 48 h	(Down)	p53,	Upregulation of miR-132 via activating the SIRT1/p53 axis could induce PD.	Qazi et al. (2021)
						NF-κB		
-	Tetrahydroxy Stilbene Glycoside (TSG)	miR-34a (Up)	-	HUVECs; pretreated with 20, 40 μg/ml TSG for 24 h	(Down)	PAI-1, p21	TSG via targeting the miR-34a/SIRT1 axis could attenuate endothelial cell premature senescence	Zhang et al. (2022)
AKI	Cisplatin (DDP)	miR-132-3p (-)	C57BL/6J mice; treated with 20 mg/kg DDP for 24, 48 h	HK-2; treated with 5 μg/ml DDP for 24, 48 h	(Down)	NF-κβ,	miR-132-3p via targeting NF-κβ by modulating SIRT1 could promote DDP-induced apoptosis in renal tubular epithelial cells	Han et al. (2021)
BLC	DDP	MST1P2 (-), miR‐133b	-	SW 780/DDP, RT4/DDP	(-)	p53,	LncRNA MST1P2/miR‐133b axis via the SIRT1/p53 axis can influence chemoresistance to DDP‐based therapy	Chen et al. (2020d)
						Caspase-3		
-	Resveratrol (RSV)	miR-155 (-)	-	N9; treated with 10 µM RSV for 1 h	(-)	AMPK, NLRP3, NF-κβ,	RSV via targeting the SIRT1/AMPK axis could inhibit NLRP3 inflammasome-induced pyroptosis and miR-155 expression in microglia	Tufekci et al. (2021)
						IL-1β, IL-18		
-	Sulforaphane (SFN)	miR-34a (Up)		HUVECs; pretreated with 1.0 μmol/l SFN for 4, 8, 12 h	(-)	Nrf2,	SFN via modulating the miR-34a/SIRT1 axis by upregulating Nrf2 could protect endothelial cells from oxidative stress	Li et al. (2021d)
						ARE		
DN	Liraglutide (LRG)	miR-34a (-)	SD rats; treated with 6 mg LRG subcutaneously for 12 weeks	-	(-)	AST, ALT, HIF-1α,	LRG via targeting the miR-34a/SIRT1 axis could regulate kidney and liver in DN rats	Xiao et al. (2021)
						Egr-1,		
						TGF-β1		

## Discussion

SIRT1 has a role as a deacetylase and is able to deacetylate a range of substrates. Thus, it participates in the regulation of a wide array of physiological processes such as gene expression, metabolic pathways and aging (Haigis and Guarente, 2006; Michan and Sinclair, 2007). This protein has functional interactions with lncRNAs, miRNAs and circRNAs. In fact, a complicated network exists between these non-coding RNAs and SIRT1. Hsa_circ_0115355/miR-326, hsa_circ_0115355/miR-487a-3p, HIPK3/miR-145, hsa_circ_0044235/miR-135b-5p, circ_0000296/miR-194-5p, circ_0001103/miR-375, CIDN/miR-34a-5p, NOP10/miR-204, circ-0082374/miR-324 are examples of circRNA/miRNA pairs that interact with SIRT1. Similarly, GAS5/miR-222-3p, GAS5/miR-579-3p, GAS5/miR-34a, MCM3AP-AS1/miR-138-5p, TUG1/miR-9-5p, TUG1/miR-29c-3p, TUG1/miR-204, SNHG8/miR‐425‐5p, SNHG7/miR-9, SNHG7/miR-34a, SNHG15/miR-141, SNHG10/miR-543, Oip5-as1/miR-29a, ILF3-AS1/miR-212-3p, ANRIL/miR-7-5p, UCA1/miR-204 and KCNQ1OT1/miR-124 are lncRNA/miRNA pairs that regulate expression of SIRT1 in different contexts. These interactions are possibly involved in the pathoetiology of a number of human disorders such as sepsis, cardiomyopathy, heart failure, non-alcoholic fatty liver disease, chronic hepatitis, cardiac fibrosis, myocardial ischemia/reperfusion injury, diabetes, ischemic stroke, immune-related disorders and cancers. In cancers, SIRT1-interacting non-coding RNAs not only affect cell proliferation but also regulate stemness and immunosuppressive responses in the tumor niche.

SIRT1 is a potential target for design of novel therapies. Most importantly, a number of drugs used for treatment of diverse asthma, sepsis, liver injury, insulin resistance, postmenopausal osteoporosis, Parkinson’s disease, diabetic nephropathy and cancers exert their effects through modulation of non-coding RNAs/SIRT1 axis. Thus, identification of the interactions between non-coding RNAs and SIRT1 has practical significance in design of novel therapeutic strategies for diverse disorders. Remarkably, non-coding RNAs that modulate expression of SIRT1 are putative modulators of the response of patients to different drugs.

## Figures and Tables

**Table:** 

**ANT**	Adjacent normal tissue
**PPARα**	peroxisome proliferators-activated receptor *a*
**CTRP6**	C1q/Tumor Necrosis Factor‐Related Protein‐6
**HMGCoAR**	β-Hydroxy *ß*-Methylglutaryl-CoA Reductase
**hUC-MSCs**	Human Umbilical Cord-Derived Mesenchymal Stem Cell
**HBE**	Human Bronchial Epithelial
**hTERT RPE-1**	Human Retinal Pigment Epithelial Cell Line
**FLSs**	Fibroblast-Like Synoviocytes
**PBMCs**	Human Peripheral Blood Mononuclear Cells
**BMSCs**	Human Bone Marrow Mesenchymal Stem Cells
**ALP**	Alkaline Phosphatase
**MPO**	Myeloperoxidase
**CECs**	Cardiac Endothelial Cells
**CFs**	Cardiac Fibroblasts
**CMs**	Cardiomyocytes
**CGRP**	Calcitonin Generated Peptide
**PGE2**	Prostaglandin E2
**BMEC**	Microvascular Endothelial Cell
**HASMCs**	Human Aortic Smooth Muscle Cells
**HIF-1α**	Hypoxia-Inducible Factor-1 *a*
**Egr-1**	Early Growth Response-1
**UA**	Uric Acid
**UREA**	Urea
**Nrf2**	Nuclear Factor Erythroid-2-Related Factor 2
**ARE**	Antioxidant Response Element
**NRVMs**	Neonatal Rats Ventricular Myocytes
**BMSCs**	Bone Marrow Mesenchymal Stem Cells
**hRMEC**	Human Retinal Microvascular Endothelial Cells
**CUMS**	Chronic Unpredictable Mild Stress
**HBDH**	Hydroxybutyratse Dehydrogenase
**CK-MB**	Creatine Kinase MB Activity
**NHSF**	Normal Human Skin Fibroblast
**RA**	Rheumatoid Arthritis
**AS**	Atherosclerosis
**SARI**	Sepsis-associated renal injury
**AML**	Pediatric acute myeloid leukemia
**HCC**	Hepatocellular carcinoma
**NSCLC**	Non-small cell lung cancer
**I/R**	Ischemia-reperfusion
**NAFLD**	Non-Alcoholic Fatty Liver Disease
**CHC**	Chronic Hepatitis C
**SCMP**	Septic Cardiomyopathy
**CHF**	Congestive Heart Failure
**HF**	Heart Failure
**MI**	Myocardial Injury
**AMI**	Acute Myocardial Infarction
**AKI**	Osteoarthritis (OA), Acute Kidney Injury
**DN**	Diabetic Nephropathy
**DR**	Diabetic Retinopathy
**SCI**	Spinal Cord Injury
**CCIS**	Chronic Constriction Injury of Sciatic Nerve
**MDD**	Major Depressive Disorder
**DFUs**	Diabetic Foot Ulcers
**ALI**	Acute Lung Injury
**UUO**	Unilateral Ureteral Obstruction
**PCa**	Pancreatic Cancer
**CRC**	Colorectal Cancer
**cSCC**	Cutaneous Squamous Cell Carcinoma
**RB**	retinoblastoma
**T2DM**	Type 2 Diabetes Mellitus
**CCI**	Chronic Cerebral Ischemia
**IDD**	Intervertebral Disc Degeneration
**GC**	Gastric Cancer
**LI**	Liver Injury
**HIR**	Hepatic Insulin Resistance
**PMOP**	Postmenopausal osteoporosis
**PD**	Parkinson’s Disease
**BLC**	Bladder Cancer

## References

[B1] Alshehri A. S. El-Kott A. F. El-Kenawy A. E. Khalifa H. S. AlRamlawy A. M. (2021). Cadmium chloride induces non-alcoholic fatty liver disease in rats by stimulating miR-34a/SIRT1/FXR/p53 axis. Sci. Total Environ. 784, 147182. 10.1016/j.scitotenv.2021.147182 34088068

[B2] Asensio-Lopez M. C. Sassi Y. Soler F. Fernandez del Palacio M. J. Pascual-Figal D. Lax A. (2021). The miRNA199a/SIRT1/P300/Yy1/sST2 signaling axis regulates adverse cardiac remodeling following MI. Sci. Rep. 11 (1), 3915–4014. 10.1038/s41598-021-82745-9 33594087 PMC7887255

[B3] Bai Y. Chen K. Zhan J. Wu M. (2020). miR-122/SIRT1 axis regulates chondrocyte extracellular matrix degradation in osteoarthritis. Biosci. Rep. 40 (6), 20191908. 10.1042/BSR20191908 PMC7308613 32395770

[B4] Bradbury C. Khanim F. Hayden R. Bunce C. White D. Drayson M. (2005). Histone deacetylases in acute myeloid leukaemia show a distinctive pattern of expression that changes selectively in response to deacetylase inhibitors. Leukemia 19 (10), 1751–1759. 10.1038/sj.leu.2403910 16121216

[B5] Chen B. Wu L. Cao T. Zheng H-M. He T. (2020b). MiR-221/SIRT1/Nrf2 signal axis regulates high glucose induced apoptosis in human retinal microvascular endothelial cells. BMC Ophthalmol. 20 (1), 300–310. 10.1186/s12886-020-01559-x 32698791 PMC7374880

[B6] Chen J. Li Y. Li Z. Cao L. (2020d). LncRNA MST1P2/miR-133b axis affects the chemoresistance of bladder cancer to cisplatin-based therapy via Sirt1/p53 signaling. J. Biochem. Mol. Toxicol. 34 (4), e22452. 10.1002/jbt.22452 32052927

[B7] Chen L. Xie W. Wang L. Zhang X. Liu E. Kou Q. (2020a). MiRNA-133a aggravates inflammatory responses in sepsis by targeting SIRT1. Int. Immunopharmacol. 88, 106848. 10.1016/j.intimp.2020.106848 32771944

[B8] Chen S. Luo Z. Chen X. (2021). Hsa_circ_0044235 regulates the pyroptosis of rheumatoid arthritis via MiR-135b-5p-SIRT1 axis. Cell Cycle 20 (12), 1107–1121. 10.1080/15384101.2021.1916272 34097558 PMC8265814

[B9] Chen Z. He M. Chen J. Li C. Zhang Q. (2020c). Long non-coding RNA SNHG7 inhibits NLRP3-dependent pyroptosis by targeting the miR-34a/SIRT1 axis in liver cancer. Oncol. Lett. 20 (1), 893–901. 10.3892/ol.2020.11635 32566017 PMC7285900

[B10] Dai Y. Ma X. Zhang J. Yu S. Zhu Y. Wang J. (2022). hsa_circ_0115355 promotes pancreatic β-cell function in patients with type 2 diabetes through the miR-145/SIRT1 axis. J. Clin. Laboratory Analysis 36, e24583. 10.1002/jcla.24583 PMC9396171 35778952

[B11] Ding S. Liu D. Wang L. Wang G. Zhu Y. (2020). Inhibiting microRNA-29a protects myocardial ischemia-reperfusion injury by targeting SIRT1 and suppressing oxidative stress and NLRP3-mediated pyroptosis pathway. J. Pharmacol. Exp. Ther. 372 (1), 128–135. 10.1124/jpet.119.256982 31481517

[B12] Dong W. Zhang H. Zhao C. Luo Y. Chen Y. (2021). Silencing of miR-150-5p ameliorates diabetic nephropathy by targeting SIRT1/p53/AMPK pathway. Front. physiology 12, 624989. 10.3389/fphys.2021.624989 PMC8064124 33897448

[B13] Fu Q. Pan H. Tang Y. Rong J. Zheng Z. (2021). MiR-200a-3p aggravates DOX-induced cardiotoxicity by targeting PEG3 through SIRT1/NF-κB signal pathway. Cardiovasc. Toxicol. 21 (4), 302–313. 10.1007/s12012-020-09620-3 33638775

[B14] Ghafouri-Fard S. Hussen B. M. Badrlou E. Abak A. Taheri M. (2021a). MicroRNAs as important contributors in the pathogenesis of colorectal cancer. Biomed. Pharmacother. 140, 111759. 10.1016/j.biopha.2021.111759 34091180

[B15] Ghafouri-Fard S. Khoshbakht T. Hussen B. M. Sarfaraz S. Taheri M. Ayatollahi S. A. (2022). Circ_CDR1as: A circular RNA with roles in the carcinogenesis. Pathology - Res. Pract. 236, 153968. 10.1016/j.prp.2022.153968 35667198

[B16] Ghafouri-Fard S. Taheri M. Hussen B. M. Vafaeimanesh J. Abak A. Vafaee R. (2021b). Function of circular RNAs in the pathogenesis of colorectal cancer. Biomed. Pharmacother. 140, 111721. 10.1016/j.biopha.2021.111721 34015582

[B17] Gu X-X. Xu X-X. Liao H-H. Wu R-N. Huang W-M. Cheng L-X. (2021). Dexmedetomidine hydrochloride inhibits hepatocyte apoptosis and inflammation by activating the lncRNA TUG1/miR-194/SIRT1 signaling pathway. J. Inflamm. 18 (1), 20–12. 10.1186/s12950-021-00287-3 PMC8157629 34039367

[B18] Haigis M. C. Guarente L. P. (2006). Mammalian sirtuins—Emerging roles in physiology, aging, and calorie restriction. Genes and Dev. 20 (21), 2913–2921. 10.1101/gad.1467506 17079682

[B19] Han S. Lin F. Ruan Y. Zhao S. Yuan R. Ning J. (2021). miR-132-3p promotes the cisplatin-induced apoptosis and inflammatory response of renal tubular epithelial cells by targeting SIRT1 via the NF-κB pathway. Int. Immunopharmacol. 99, 108022. 10.1016/j.intimp.2021.108022 34339961

[B20] Hao R. Song X. Sun-Waterhouse D. Tan X. Li F. Li D. (2021). Mir-34a/sirt1/p53 signaling pathway contributes to cadmium-induced nephrotoxicity: A preclinical study in mice. Environ. Pollut. 282, 117029. 10.1016/j.envpol.2021.117029 33823310

[B21] Huang K. Yang C. Zheng J. Liu X. Liu J. Che D. (2021b). Effect of circular RNA, mmu_circ_0000296, on neuronal apoptosis in chronic cerebral ischaemia via the miR-194-5p/Runx3/Sirt1 axis. Cell Death Discov. 7 (1), 124–215. 10.1038/s41420-021-00507-y 34052838 PMC8164632

[B22] Huang L. Cai H-A. Zhang M-S. Liao R-Y. Huang X. Hu F-D. (2021a). Ginsenoside Rg1 promoted the wound healing in diabetic foot ulcers via miR-489–3p/Sirt1 axis. J. Pharmacol. Sci. 147 (3), 271–283. 10.1016/j.jphs.2021.07.008 34507636

[B23] Huffman D. M. Grizzle W. E. Bamman M. M. Kim J-S. Eltoum I. A. Elgavish A. (2007). SIRT1 is significantly elevated in mouse and human prostate cancer. Cancer Res. 67 (14), 6612–6618. 10.1158/0008-5472.CAN-07-0085 17638871

[B24] Hussen B. M. Hidayat H. J. Ghafouri-Fard S. (2022b). Identification of expression of CCND1-related lncRNAs in breast cancer. Pathology - Res. Pract. 236, 154009. 10.1016/j.prp.2022.154009 35803208

[B25] Hussen B. M. Hidayat H. J. Salihi A. Sabir D. K. Taheri M. Ghafouri-Fard S. (2021). MicroRNA: A signature for cancer progression. Biomed. Pharmacother. 138, 111528. 10.1016/j.biopha.2021.111528 33770669

[B26] Hussen B. M. Salihi A. Abdullah S. T. Rasul M. F. Hidayat H. J. Hajiesmaeili M. (2022a). Signaling pathways modulated by miRNAs in breast cancer angiogenesis and new therapeutics. Pathology - Res. Pract. 230, 153764. 10.1016/j.prp.2022.153764 35032831

[B27] Kang M. Ji F. Sun X. Liu H. Zhang C. (2021). LncRNA SNHG15 promotes oxidative stress damage to regulate the occurrence and development of cerebral ischemia/reperfusion injury by targeting the miR-141/SIRT1 axis. J. Healthc. Eng. 2021, 6577799. 10.1155/2021/6577799 34868528 PMC8641992

[B28] Li C. Wang F. Miao P. Yan L. Liu S. Wang X. (2020b). miR-138 increases depressive-like behaviors by targeting SIRT1 in hippocampus. Neuropsychiatric Dis. Treat. 16, 949–957. 10.2147/NDT.S237558 PMC7154038 32308399

[B29] Li F. Zhang L. Xue H. Xuan J. Rong S. Wang K. (2021a). SIRT1 alleviates hepatic ischemia-reperfusion injury via the miR-182-mediated XBP1/NLRP3 pathway. Mol. Therapy-Nucleic Acids. 23, 1066–1077. 10.1016/j.omtn.2020.11.015 PMC7887305 33664991

[B30] Li H. Chou P. Du F. Sun L. Liu J. Wang W. (2021b). Depleting microRNA-183-3p improves renal tubulointerstitial fibrosis after acute kidney injury via SIRT1/PUMA/FOXO3a deacetylation. Life Sci. 269, 119017. 10.1016/j.lfs.2021.119017 33450262

[B31] Li T. Jiang H. Li Y. Zhao X. Ding H. (2021c). Estrogen promotes lncRNA H19 expression to regulate osteogenic differentiation of BMSCs and reduce osteoporosis via miR-532-3p/SIRT1 axis. Mol. Cell. Endocrinol. 527, 111171. 10.1016/j.mce.2021.111171 33577975

[B32] Li T. Pang Q. Liu Y. Bai M. Peng Y. Zhang Z. (2021d). Sulforaphane protects human umbilical vein endothelial cells from oxidative stress via the miR-34a/SIRT1 axis by upregulating nuclear factor erythroid-2-related factor 2. Exp. Ther. Med. 21 (3), 186. 10.3892/etm.2021.9617 33488795 PMC7812584

[B33] Li X. Zhang W. Xu K. Lu J. (2020a). miR-34a promotes liver fibrosis in patients with chronic hepatitis via mediating Sirt1/p53 signaling pathway. Pathology-Research Pract. 216 (5), 152876. 10.1016/j.prp.2020.152876 32089410

[B34] Liang Y. Li E. Zhang H. Zhang L. Tang Y. Wanyan Y. (2020). Silencing of lncRNA UCA1 curbs proliferation and accelerates apoptosis by repressing SIRT1 signals by targeting miR-204 in pediatric AML. J. Biochem. Mol. Toxicol. 34 (3), e22435. 10.1002/jbt.22435 31916649

[B35] Liang Y. Liu H. Zhu J. Song N. Lu Z. Fang Y. (2021). Inhibition of p53/miR-34a/SIRT1 axis ameliorates podocyte injury in diabetic nephropathy. Biochem. Biophysical Res. Commun. 559, 48–55. 10.1016/j.bbrc.2021.04.025 33932899

[B36] Ling H. Li Q. Duan Z-P. Wang Y-J. Hu B-Q. Dai X-G. (2021). LncRNA GAS5 inhibits miR-579-3p to activate SIRT1/PGC-1α/Nrf2 signaling pathway to reduce cell pyroptosis in sepsis-associated renal injury. Am. J. Physiology-Cell Physiology 321 (7), C117–C133. 10.1152/ajpcell.00394.2020 34010066

[B37] Liu M. Zhang Y. Cao X. Shi T. Yan Y. (2022a). miR-197 participates in lipopolysaccharide-induced cardiomyocyte injury by modulating SIRT1. Cardiol. Res. Pract. 2022, 7687154. 10.1155/2022/7687154 35223094 PMC8872679

[B38] Liu Y. Zhang M. Zhang H. Qian X. Luo L. He Z. (2022b). Cancer metastases from lung adenocarcinoma disappeared after molecular targeted therapy: A successfully clinical treatment experience. Int. Archives Allergy Immunol. 183 (5), 539–546. 10.2147/PGPM.S367978 PMC9150760 35651533

[B39] Lu R-H. Xiao Z-Q. Zhou J-D. Yin C-Q. Chen Z-Z. Tang F-J. (2020). MiR-199a-5p represses the stemness of cutaneous squamous cell carcinoma stem cells by targeting Sirt1 and CD44ICD cleavage signaling. Cell Cycle 19 (1), 1–14. 10.1080/15384101.2019.1689482 31809227 PMC6927716

[B40] Ma W. Zhang W. Cui B. Gao J. Liu Q. Yao M. (2021). Functional delivery of lncRNA TUG1 by endothelial progenitor cells derived extracellular vesicles confers anti-inflammatory macrophage polarization in sepsis via impairing miR-9-5p-targeted SIRT1 inhibition. Cell death Dis. 12 (11), 1056–1110. 10.1038/s41419-021-04117-5 34743197 PMC8572288

[B41] Meng F. Yang M. Chen Y. Chen W. Wang W. (2021). miR-34a induces immunosuppression in colorectal carcinoma through modulating a SIRT1/NF-κB/B7-H3/TNF-α axis. Immunotherapy 70 (8), 2247–2259. 10.1007/s00262-021-02862-2 PMC10991903 33492448

[B42] Michan S. Sinclair D. (2007). Sirtuins in mammals: Insights into their biological function. Biochem. J. 404 (1), 1–13. 10.1042/BJ20070140 17447894 PMC2753453

[B43] Mo Y. Liu B. Qiu S. Wang X. Zhong L. Han X. (2020). Down-regulation of microRNA-34c-5p alleviates neuropathic pain via the SIRT1/STAT3 signaling pathway in rat models of chronic constriction injury of sciatic nerve. J. Neurochem. 154 (3), 301–315. 10.1111/jnc.14998 32126145

[B44] Niu X. Pu S. Ling C. Xu J. Wang J. Sun S. (2020). lncRNA Oip5-as1 attenuates myocardial ischaemia/reperfusion injury by sponging miR-29a to activate the SIRT1/AMPK/PGC1α pathway. Cell Prolif. 53 (6), e12818. 10.1111/cpr.12818 32468629 PMC7309946

[B45] Pillarisetti S. (2008). A review of Sirt1 and Sirt1 modulators in cardiovascular and metabolic diseases. Recent Pat. Cardiovasc. Drug Discov. Discontin. 3 (3), 156–164. 10.2174/157489008786263989 18991791

[B46] Purushotham A. Schug T. T. Xu Q. Surapureddi S. Guo X. Li X. (2009). Hepatocyte-specific deletion of SIRT1 alters fatty acid metabolism and results in hepatic steatosis and inflammation. Cell metab. 9 (4), 327–338. 10.1016/j.cmet.2009.02.006 19356714 PMC2668535

[B47] Qazi T. J. Lu J. Duru L. Zhao J. Qing H. (2021). Upregulation of mir-132 induces dopaminergic neuronal death via activating SIRT1/P53 pathway. Neurosci. Lett. 740, 135465. 10.1016/j.neulet.2020.135465 33166640

[B48] Qi M. He L. Ma X. Li Z. (2020). MiR-181a-5p is involved in the cardiomyocytes apoptosis induced by hypoxia–reoxygenation through regulating SIRT1. Biosci. Biotechnol. Biochem. 84 (7), 1353–1361. 10.1080/09168451.2020.1750943 32290769

[B49] Qu L. H. Hong X. Zhang Y. Cong X. Xiang R. L. Mei M. (2021). C1q/tumor necrosis factor-related protein-6 attenuates TNF-α-induced apoptosis in salivary acinar cells via AMPK/SIRT1-modulated miR-34a-5p expression. J. Cell. Physiology 236 (8), 5785–5800. 10.1002/jcp.30262 33400820

[B50] Rahman S. Islam R. (2011). Mammalian Sirt1: Insights on its biological functions. Cell Commun. Signal. 9 (1), 11–18. 10.1186/1478-811X-9-11 21549004 PMC3103488

[B51] Sabaie H. Salkhordeh Z. Asadi M. R. Ghafouri-Fard S. Amirinejad N. Askarinejad Behzadi M. (2021). Long non-coding RNA- associated competing endogenous RNA axes in T-cells in multiple sclerosis. Front. Immunol. 12, 770679. 10.3389/fimmu.2021.770679 34956196 PMC8696673

[B52] Sayad A. Najafi S. Hussen B. M. Jamali E. Taheri M. Ghafouri-Fard S. (2022). The role of circular RNAs in pancreatic cancer: New players in tumorigenesis and potential biomarkers. Pathology - Res. Pract. 232, 153833. 10.1016/j.prp.2022.153833 35272115

[B53] Shi J. Cao F. Chang Y. Xin C. Jiang X. Xu J. (2021). Long non-coding RNA MCM3AP-AS1 protects chondrocytes ATDC5 and CHON-001 from IL-1β-induced inflammation via regulating miR-138-5p/SIRT1. Bioengineered 12 (1), 1445–1456. 10.1080/21655979.2021.1905247 33942704 PMC8806229

[B54] Shu L. Zhang W. Huang C. Huang G. Su G. Xu J. (2020). lncRNA ANRIL protects H9c2 cells against hypoxia‐induced injury through targeting the miR-7-5p/SIRT1 axis. J. Cell. physiology 235 (2), 1175–1183. 10.1002/jcp.29031 31264206

[B55] Stünkel W. Peh B. K. Tan Y. C. Nayagam V. M. Wang X. Salto-Tellez M. (2007). Function of the SIRT1 protein deacetylase in cancer. Biotechnol. J. Healthc. Nutr. Technol. 2 (11), 1360–1368. 10.1002/biot.200700087 17806102

[B56] Sui M. Jiang X. Sun H. Liu C. Fan Y. (2021). Berberine ameliorates hepatic insulin resistance by regulating microRNA-146b/SIRT1 pathway. Diabetes, metabolic syndrome Obes. targets Ther. 14, 2525–2537. 10.2147/DMSO.S313068 PMC8187038 34113144

[B57] Sun G. Yang L. Wei S. Jin H. Li B. Li H. (2021). miR-425 regulates lipophagy via SIRT1 to promote sorafenib resistance in liver cancer. Oncol. Lett. 22 (4), 695–710. 10.3892/ol.2021.12956 34457050 PMC8358621

[B58] Tian J. Liu Y. Wang Z. Zhang S. Yang Y. Zhu Y. (2021). LncRNA Snhg8 attenuates microglial inflammation response and blood–brain barrier damage in ischemic stroke through regulating miR-425-5p mediated SIRT1/NF-κB signaling. J. Biochem. Mol. Toxicol. 35 (5), e22724. 10.1002/jbt.22724 33491845

[B59] Tufekci K. U. Eltutan B. I. Isci K. B. Genc S. (2021). Resveratrol inhibits NLRP3 inflammasome-induced pyroptosis and miR-155 expression in microglia through Sirt1/AMPK pathway. Neurotox. Res. 39 (6), 1812–1829. 10.1007/s12640-021-00435-w 34739715

[B60] Vaziri H. Dessain S. K. Eaton E. N. Imai S-I. Frye R. A. Pandita T. K. (2001). hSIR2SIRT1 functions as an NAD-dependent p53 deacetylase. Cell 107 (2), 149–159. 10.1016/s0092-8674(01)00527-x 11672523

[B61] Wang B. Li B. Si T. (2020). Knockdown of circ0082374 inhibits cell viability, migration, invasion and glycolysis in glioma cells by miR-326/SIRT1. Brain Res. 1748, 147108. 10.1016/j.brainres.2020.147108 32896523

[B62] Wang C. Guo X. Wang Y. Wang H. (2021c). Silencing of miR-324-5p alleviates rat spinal cord injury by Sirt1. Neurosci. Res. 173, 34–43. 10.1016/j.neures.2021.05.010 34051279

[B63] Wang H. He F. Liang B. Jing Y. Zhang P. Liu W. (2021g). LincRNA-p21 alleviates atherosclerosis progression through regulating the miR-221/SIRT1/Pcsk9 axis. J. Cell. Mol. Med. 25 (19), 9141–9153. 10.1111/jcmm.16771 34541816 PMC8500963

[B64] Wang R. Xu Y. Niu X. Fang Y. Guo D. Chen J. (2021b). miR-22 inhibition alleviates cardiac dysfunction in doxorubicin-induced cardiomyopathy by targeting the sirt1/PGC-1α pathway. Front. Physiology 12, 646903. 10.3389/fphys.2021.646903 PMC8047466 33868015

[B65] Wang R. Xu Y. Zhang W. Fang Y. Yang T. Zeng D. (2021a). Inhibiting miR-22 alleviates cardiac dysfunction by regulating Sirt1 in septic cardiomyopathy. Front. Cell Dev. Biol. 9, 650666. 10.3389/fcell.2021.650666 33869205 PMC8047209

[B66] Wang S. Yi P. Wang N. Song M. Li W. Zheng Y. (2021h). LncRNA TUG1/miR-29c-3p/SIRT1 axis regulates endoplasmic reticulum stress-mediated renal epithelial cells injury in diabetic nephropathy model *in vitro* . PloS one 16 (6), e0252761. 10.1371/journal.pone.0252761 34097717 PMC8183992

[B67] Wang Y. Yang Z. Zhang K. Wan Y. Zhou Y. Yang Z. (2021d). miR-135a-5p inhibitor protects glial cells against apoptosis via targeting SIRT1 in epilepsy. Exp. Ther. Med. 21 (5), 431–438. 10.3892/etm.2021.9848 33747170 PMC7967866

[B68] Wang Z. Chen R. Xu Z. Ru W. Tian H. Yang F. (2021e). MiR-155-5p promotes renal interstitial fibrosis in obstructive nephropathy via inhibiting SIRT1 signaling pathway. J. Recept. Signal Transduct. 41 (5), 466–475. 10.1080/10799893.2020.1825491 32985331

[B69] Wang Z. Shi D. Zhang N. Yuan T. Tao H. (2021f). MiR-217 promotes endothelial cell senescence through the SIRT1/p53 signaling pathway. J. Mol. Histology 52 (2), 257–267. 10.1007/s10735-020-09945-x 33392891

[B70] Wei Y-J. Wang J-F. Cheng F. Xu H-J. Chen J-J. Xiong J. (2021). miR-124-3p targeted SIRT1 to regulate cell apoptosis, inflammatory response, and oxidative stress in acute myocardial infarction in rats via modulation of the FGF21/CREB/PGC1α pathway. J. physiology Biochem. 77 (4), 577–587. 10.1007/s13105-021-00822-z 34146302

[B71] Wu L. Zhang G. Guo C. Zhao X. Shen D. Yang N. (2020). MiR-128-3p mediates TNF-α-induced inflammatory responses by regulating Sirt1 expression in bone marrow mesenchymal stem cells. Biochem. biophysical Res. Commun. 521 (1), 98–105. 10.1016/j.bbrc.2019.10.083 31635801

[B72] Wu P. Yu X. Peng Y. Wang Q-L. Deng L-T. Xing W. (2021b). Ginsenoside Rg3 alleviates septic liver injury by regulating the lncRNA TUG1/miR-200c-3p/SIRT1 axis. J. Inflamm. 18 (1), 31–13. 10.1186/s12950-021-00296-2 PMC8686388 34930287

[B73] Wu Z. Chen J. Zhao W. Zhuo C. H. Chen Q. (2021a). Inhibition of miR-181a attenuates sepsis-induced inflammation and apoptosis by activating Nrf2 and inhibiting NF-κB pathways via targeting SIRT1. Kaohsiung J. Med. Sci. 37 (3), 200–207. 10.1002/kjm2.12310 33058411 PMC11896183

[B74] Xiang Q. Kang L. Wang J. Liao Z. Song Y. Zhao K. (2020). CircRNA-CIDN mitigated compression loading-induced damage in human nucleus pulposus cells via miR-34a-5p/SIRT1 axis. EBioMedicine 53, 102679. 10.1016/j.ebiom.2020.102679 32114390 PMC7044714

[B75] Xiao S. Yang Y. Liu Y-T. Zhu J. (2021). Liraglutide regulates the kidney and liver in diabetic nephropathy rats through the miR-34a/SIRT1 pathway. J. Diabetes Res. 2021, 8873956. 10.1155/2021/8873956 33880382 PMC8046563

[B76] Xu H. Zhang J. Shi X. Li X. Zheng C. (2021a). NF-κB inducible miR-30b-5p aggravates joint pain and loss of articular cartilage via targeting SIRT1-FoxO3a-mediated NLRP3 inflammasome. Aging (Albany NY) 13 (16), 20774–20792. 10.18632/aging.203466 34455406 PMC8436920

[B77] Xu J. Wang X. Wang W. Zhang L. Huang P. (2021b). Candidate oncogene circularNOP10 mediates gastric cancer progression by regulating miR-204/SIRT1 pathway. J. Gastrointest. Oncol. 12 (4), 1428–1443. 10.21037/jgo-21-422 34532100 PMC8421869

[B78] Yamamoto H. Schoonjans K. Auwerx J. (2007). Sirtuin functions in health and disease. Mol. Endocrinol. 21 (8), 1745–1755. 10.1210/me.2007-0079 17456799

[B79] Yang Y. Li L. (2021). Depleting microRNA-146a-3p attenuates lipopolysaccharide-induced acute lung injury via up-regulating SIRT1 and mediating NF-κB pathway. J. Drug Target. 29 (4), 420–429. 10.1080/1061186X.2020.1850738 33185125

[B80] Yang Y-L. Wang P-W. Wang F-S. Lin H-Y. Huang Y-H. (2020). miR-29a modulates GSK3β/SIRT1-linked mitochondrial proteostatic stress to ameliorate mouse non-alcoholic steatohepatitis. Int. J. Mol. Sci. 21 (18), 6884. 10.3390/ijms21186884 32961796 PMC7555728

[B81] Yang Z. Lin S-D. Zhan F. Liu Y. Zhan Y-W. (2021). LncRNA GAS5 alleviates rheumatoid arthritis through regulating miR-222-3p/Sirt1 signalling axis. Autoimmunity 54 (1), 13–22. 10.1080/08916934.2020.1846183 33215529

[B82] Yin Q-H. Zhou Y. Li Z-Y. (2021). miR-373 suppresses cell proliferation and apoptosis via regulation of SIRT1/PGC-1α/NRF2 axis in pancreatic cancer. Cell J. (Yakhteh) 23 (2), 199–210. 10.22074/cellj.2021.7038 PMC8181315 34096221

[B83] Yuan T. Zhang L. Yao S. Deng S. Y. Liu J. Q. (2020). miR-195 promotes LPS-mediated intestinal epithelial cell apoptosis via targeting SIRT1/eIF2a. Int. J. Mol. Med. 45 (2), 510–518. 10.3892/ijmm.2019.4431 31894250 PMC6984803

[B84] Zhan H. Huang F. Niu Q. Jiao M. Han X. Zhang K. (2021). Downregulation of miR-128 ameliorates Ang II-induced cardiac remodeling via SIRT1/PIK3R1 multiple targets. Oxidative Med. Cell. Longev. 2021, 8889195. 10.1155/2021/8889195 PMC8505057 34646427

[B85] Zhang H. Yang X. Xu Y. Li H. (2021d). KCNQ1OT1 regulates the retinoblastoma cell proliferation, migration and SIRT1/JNK signaling pathway by targeting miR-124/SP1 axis. Biosci. Rep. 41 (1), 20201626. 10.1042/BSR20201626 PMC7805023 33345272

[B86] Zhang H. Zhang X. M. Zong Dd Xy Ji Jiang H. Zhang F. Z. (2021a). miR-34a-5p up-regulates the IL-1β/COX2/PGE2 inflammation pathway and induces the release of CGRP via inhibition of SIRT1 in rat trigeminal ganglion neurons. FEBS Open bio 11 (1), 300–311. 10.1002/2211-5463.13027 PMC7780114 33155431

[B87] Zhang H. G. Wang F. J. Wang Y. Zhao Z. X. Qiao P. F. (2021c). lncRNA GAS5 inhibits malignant progression by regulating macroautophagy and forms a negative feedback regulatory loop with the miR-34a/mTOR/SIRT1 pathway in colorectal cancer. Oncol. Rep. 45 (1), 202–216. 10.3892/or.2020.7825 33416133 PMC7709827

[B88] Zhang J. Yang Z. Fang K. Shi Z. Ren D. Sun J. (2020c). Long noncoding RNA ILF3-AS1 regulates myocardial infarction via the miR-212-3p/SIRT1 axis and PI3K/Akt signaling pathway. Eur. Rev. Med. Pharmacol. Sci. 24 (5), 2647–2658. 10.26355/eurrev_202003_20534 32196615

[B89] Zhang L. Guo Y. Shi S. Zhuge Y. Chen N. Ding Z. (2022). Tetrahydroxy stilbene glycoside attenuates endothelial cell premature senescence induced by H2O2 through the microRNA-34a/SIRT1 pathway. Sci. Rep. 12 (1), 1708–8. 10.1038/s41598-022-05804-9 35105933 PMC8807705

[B90] Zhang M. Cao M. Kong L. Liu J. Wang Y. Song C. (2020a). MiR-204-5p promotes lipid synthesis in mammary epithelial cells by targeting SIRT1. Biochem. Biophysical Res. Commun. 533 (4), 1490–1496. 10.1016/j.bbrc.2020.10.056 33333715

[B91] Zhang M. Mou L. Liu S. Sun F. Gong M. (2021b). Circ_0001103 alleviates IL-1β-induced chondrocyte cell injuries by upregulating SIRT1 via targeting miR-375. Clin. Immunol. 227, 108718. 10.1016/j.clim.2021.108718 33819576

[B92] Zhang Y. Ma Y. Gu M. Peng Y. (2020d). lncRNA TUG1 promotes the Brown remodeling of white adipose tissue by regulating miR-204-targeted SIRT1 in diabetic mice. Int. J. Mol. Med. 46 (6), 2225–2234. 10.3892/ijmm.2020.4741 33125086

[B93] Zhang Z. Nong L. Chen M-L. Gu X-L. Zhao W-W. Liu M-H. (2020b). Long noncoding RNA SNHG10 sponges miR-543 to upregulate tumor suppressive SIRT1 in nonsmall cell lung cancer. Cancer Biotherapy Radiopharm. 35 (10), 771–775. 10.1089/cbr.2019.3334 32319822

[B94] Zheng M. Tan J. Liu X. Jin F. Lai R. Wang X. (2021). miR-146a-5p targets Sirt1 to regulate bone mass. Bone Rep. 14, 101013. 10.1016/j.bonr.2021.101013 33855130 PMC8024884

[B95] Zhou F. Wang Y-K. Zhang C-G. Wu B-Y. (2021). miR-19a/b-3p promotes inflammation during cerebral ischemia/reperfusion injury via SIRT1/FoxO3/SPHK1 pathway. J. neuroinflammation 18 (1), 122–213. 10.1186/s12974-021-02172-5 34051800 PMC8164774

[B96] Zhou T. Wang S. Lu K. Yin C. (2020). Long non-coding RNA SNHG7 alleviates oxygen and glucose deprivation/reoxygenation-induced neuronal injury by modulating miR-9/SIRT1 Axis in PC12 cells: Potential role in ischemic stroke. Neuropsychiatric Dis. Treat. 16, 2837–2848. 10.2147/NDT.S273421 PMC7700012 33262598

[B97] Zhuang L. Wang Z. Hu X. Yang Q. Pei X. Jin G. (2021). CircHIPK3 alleviates high glucose toxicity to human renal tubular epithelial HK-2 cells through regulation of miR-326/miR-487a-3p/SIRT1. Targets Ther. 14, 729–740. 10.2147/DMSO.S289624 PMC7898210 33628038

